# Effect of Diacetylcurcumin Manganese Complex on Rotenone-Induced Oxidative Stress, Mitochondria Dysfunction, and Inflammation in the SH-SY5Y Parkinson’s Disease Cell Model

**DOI:** 10.3390/molecules29050957

**Published:** 2024-02-22

**Authors:** Ekanong Pirunkaset, Chantana Boonyarat, Juthamart Maneenet, Charinya Khamphukdee, Supawadee Daodee, Orawan Monthakantirat, Suresh Awale, Anake Kijjoa, Yaowared Chulikhit

**Affiliations:** 1 Graduate School of Pharmaceutical Sciences, Khon Kaen University, Khon Kaen 40002, Thailand; ekanong.p@kkumail.com; 2 Division of Pharmaceutical Chemistry, Faculty of Pharmaceutical Sciences, Khon Kaen University, Khon Kaen 40002, Thailand; chaboo@kku.ac.th (C.B.); juthamart_pp@hotmail.com (J.M.); csupawad@kku.ac.th (S.D.); oramon@kku.ac.th (O.M.); 3 Natural Drug Discovery Laboratory, Institute of Natural Medicine, University of Toyama, Toyama 930-0154, Japan; suresh@inm.u-toyama.ac.jp; 4 Division of Pharmacognosy and Toxicology, Faculty of Pharmaceutical Sciences, Khon Kaen University, Khon Kaen 40002, Thailand; charkh@kku.ac.th; 5 ICBAS-Instituto de Ciências Biomédicas Abel Salazar and CIIMAR, Universidade do Porto, 4050-313 Porto, Portugal; ankijjoa@icbas.up.pt

**Keywords:** diacetylcurcumin manganese complex, Parkinson’s disease, oxidative stress, SOD mimics, Nrf2 regulation

## Abstract

Diacetylcurcumin manganese complex (DiAc-Cp-Mn) is a diacetylcurcumin (DiAc-Cp) derivative synthesized with Mn (II) to mimic superoxide dismutase (SOD). It exhibited superior reactive oxygen species (ROS) scavenging efficacy, particularly for the superoxide radical. The present study investigated the ROS scavenging activity, neuroprotective effects, and underlying mechanism of action of DiAc-Cp-Mn in a cellular model of Parkinson’s disease. This study utilized rotenone-induced neurotoxicity in SH-SY5Y cells to assess the activities of DiAc-Cp-Mn by measuring cell viability, intracellular ROS, mitochondrial membrane potential (MMP), SOD, and catalase (CAT) activities. The mRNA expression of the nuclear factor erythroid 2 p45-related factor (Nrf2), Kelch-like ECH-associated protein 1 (Keap1), inducible nitric oxide synthase (iNOS), and Interleukin 1β (IL-1β), which are oxidative and inflammatory genes, were also evaluated to clarify the molecular mechanism. The results of the in vitro assays showed that DiAc-Cp-Mn exhibited greater scavenging activity against superoxide radicals, hydrogen peroxide, and hydroxyl radicals compared to DiAc-Cp. In cell-based assays, DiAc-Cp-Mn demonstrated greater neuroprotective effects against rotenone-induced neurotoxicity when compared to its parent compound, DiAc-Cp. DiAc-Cp-Mn maintained MMP levels, reduced intracellular ROS levels, and increased the activities of SOD and CAT by activating the Nrf2-Keap1 signaling pathway. In addition, DiAc-Cp-Mn exerted its anti-inflammatory impact by down-regulating the mRNA expression of iNOS and IL-1β that provoked neuro-inflammation. The current study indicates that DiAc-Cp-Mn protects against rotenone-induced neuronal damage by reducing oxidative stress and inflammation.

## 1. Introduction

Parkinson’s disease (PD) is the second most common progressive neurodegenerative disease after Alzheimer’s disease and the most common in people over the age of 60. PD is characterized by the loss of dopaminergic neurons in the pars compacta of the substantia nigra (SNpc). The pathological hallmark of PD is the presence of Lewy bodies (LBs) containing α-synuclein aggregates that induce oxidative stress and mitochondrial dysfunction, which, in turn, cause dopaminergic neuronal degeneration [1], resulting in motor symptoms such as resting tremor, rigidity, and bradykinesia with postural instability. Idiopathic PD shows multifactorial pathogenesis, with aging, genetic factors, and environmental factors all contributing via mechanisms including oxidative stress, mitochondrial dysfunction, neuroinflammation, protein aggregation, and proteolysis defects [2].

Reactive species include superoxide anion radicals, hydrogen peroxide (H_2_O_2_), hydroxyl radicals (HO^•^), nitric oxide (NO), and peroxynitrite anions (ONOO–) [3]. The superoxide radical (O_2_^•−^) is the primary reactive species, generated from mitochondrial electron transport chains (ETC). The sites of ROS production in the ETC are from the mitochondria complexes I and III, as they occasionally release electrons, and then electrons incompletely reduce oxygen molecules to superoxide radicals. Generally, the antioxidant defense system protects the body from reactive species-induced cellular damage through the activities of antioxidant enzymes such as SOD, CAT, and glutathione peroxidase (GPx). SOD catalyzes the conversion of the superoxide radical to H_2_O_2_, which is converted to water by the activity of CAT and GPX [4,5]. Oxidative stress is caused by the overproduction of reactive species and a deficiency in antioxidant enzymes. Excessive ROS accumulation causes neuronal cell loss and mitochondrial dysfunction, leading to deficits in energy production and the accumulation of toxic protein aggregates. These are associated with neurodegenerative disorders, including Parkinson’s disease (PD). In response to brain oxidative damage, the Nrf2/Keap1 pathway is now recognized as one of the most important endogenous defense systems. Under homeostatic conditions, the cytoplasmic protein Keap1 binds to Nrf2 and suppresses its function. Redox imbalance conditions induce the dissociation of Nrf2 from the Keap1 complex, releasing the nuclear factor that translocates into the nucleus and binds to the antioxidant response element (ARE). The active Nrf2 binds to ARE to transcriptionally activate the antioxidant genes and trigger antioxidant responses such as SOD, GPx, heme oxygenase 1 (HO-1), and NADH(P)H: quinone oxidoreductase 1 (NQO1) [6]. These antioxidant enzymes act by reducing oxidative stress. The ROS level is then regulated to the normal level.

The SH-SY5Y dopaminergic neuronal cell line is an important in vitro model for determining the mechanism of PD pathogenesis [7]. In addition, several neurotoxin-based models use compounds such as 6-hydroxydopamine (6-OHDA), 1-methyl-4-phenyl-1,2,3,6,-tetrahydropyridine (MPTP), paraquat, maneb, and rotenone to simulate PD by damaging dopaminergic neurons in the SNpc via various mechanisms. Rotenone is a natural pesticide and insecticide that easily crosses the blood-brain barrier (BBB) and cellular plasma membrane due to its high lipophilicity. Rotenone inhibits mitochondrial complex I on the respiratory electron transport chain, resulting in mitochondrial dysfunction, oxidative stress, and cell apoptosis. The rotenone PD model induces motor symptoms, non-motor symptoms, and the formation of LBs containing α-synuclein inclusions. Moreover, the rotenone PD model causes genetic mutation, which is an invaluable tool for the study of gene causation in PD [8].

**Figure 1:**
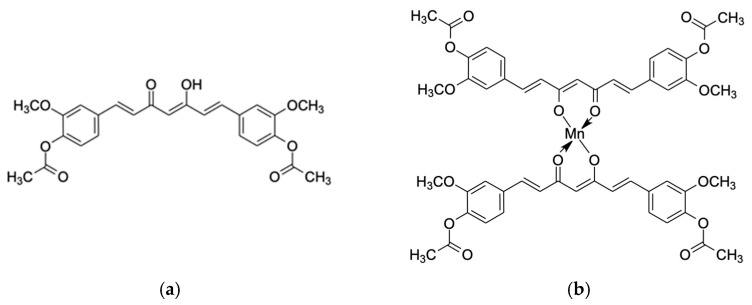
Chemical structure of (**a**) diacetylcurcumin (DiAc-Cp) and (**b**) diacetylcurcumin manganese complex (DiAc-Cp-Mn).

Diacetylcurcumin is a synthetic derivative of curcumin, where the free phenolic groups of curcumin have been derivatized by acetylation to confer greater lipophilicity and partially overcome the limited systemic bioavailability of curcumin [9]. The diacetylcurcumin manganese complex was a diacetylcurcumin derivative synthesized with Mn (II) as a low molecular weight compound with more potent superoxide radical scavenging activity [10] (Figure 1). In in vivo studies, kainic acid was able to induce seizures and neuronal cell loss and upregulate neurotoxic marker genes. Treatment with DiAc-Cp-Mn significantly decreased pyramidal cell loss in the hippocampus [11], delayed the onset but not severity of KA-induced seizures, and downregulated the expression of iNOS and cyclooxygenase-2 (COX-2) [12]. The manganese complex also showed neuroprotective effects, preventing learning and memory impairment in transient cerebral ischemic mice [13]. While these findings support the improved neuroprotective effects of the complex compared to its parent compound, the neuroprotective effects of DiAc-Cp-Mn on PD have not been elucidated.

The present study aimed to evaluate the neuroprotective effects and the underlying mechanism of action of the DiAc-Cp-Mn complexes against rotenone-induced PD in an SH-SY5Y cell model of PD.

## 2. Results

### 2.1. In Vitro Antioxidant Assays of DiAc-Cp and DiAc-Cp-Mn

The superoxide radical, hydrogen peroxide, and hydroxyl radical scavenging abilities were measured and reported in Table 1. The superoxide radical is a primary free radical that is produced continuously from the mitochondrial electron transport chain and contributes to tissue damage and various diseases. The SOD-like activity of DiAc-Cp-Mn was examined employing the nitro blue tetrazolium (NBT) assay. NBT undergoes reduction in the presence of a superoxide anion. In this assay, the capability of the interested compounds to prevent NBT reduction was assessed. The results showed that the IC_50_ superoxide radical scavenging values for DiAc-Cp, DiAc-Cp-Mn, and SOD were 211.10 ± 4.27 µM, 5.69 ± 0.05 µM, and 5.90 ± 0.20 unit/mL, respectively. The SOD-like activity of DiAc-Cp-Mn was more potent than that of its parent compound, DiAc-Cp. Hydrogen peroxide is a less-reactive ROS that is toxic to living cells at high concentrations. DiAc-Cp-Mn showed the highest hydrogen peroxide scavenging activity (IC_50_ 85.57 ± 0.32 µM), whereas the IC_50_ of DiAc-Cp was 218.02 ± 3.00 µM and the IC_50_ of trolox was 1118.18 ± 15.14 µM. In addition, the hydroxyl radical scavenging activity was also assessed. The hydroxyl radical is a highly reactive radical that induces severe damage to proteins, DNA, and lipids, leading to lipid peroxidation. The IC_50_ hydroxyl radical scavenging values for DiAc-Cp, DiAc-Cp-Mn, and trolox were 46.94 ± 1.65 µM, 15.14 ± 0.19 µM, and 99.78 ± 14.26 µM, respectively. DiAc-Cp-Mn was the most potent scavenger of hydroxyl radicals. Taken together, DiAc-Cp-Mn exhibited superior SOD and CAT-like activities and hydroxyl radical scavenging properties when compared to its parent compound.

### 2.2. Effect of DiAc-Cp and DiAc-Cp-Mn on the Viability of SH-SY5Y Cells

**Figure 2:**
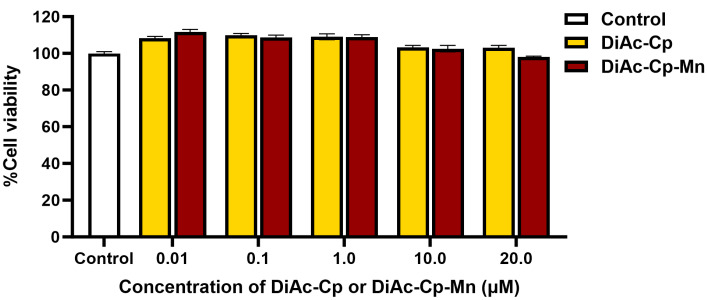
The cytotoxicity of DiAc-Cp and DiAc-Cp-Mn at 24 h in SH-SY5Y cells was determined by an MTT assay. Values are reported as the percentage of the untreated control and represent the mean ± SEM (*n* = 6).

To evaluate the effect of DiAc-Cp and DiAc-Cp-Mn on cell viability, SH-SY5Y cells were treated with various concentrations of DiAc-Cp and DiAc-Cp-Mn (0.01, 0.1, 1.0, 10.0, and 20.0 μM) for 24 h. The cell viability was determined using an MTT assay. As shown in Figure 2, treatment of SH-SY5Y cells with DiAc-Cp and DiAc-Cp-Mn at concentrations ranging from 0.01 to 20.0 μM for 24 h did not affect cell viability compared to the control group.

### 2.3. Effect of Rotenone on the Viability of SH-SY5Y Cells

**Figure 3:**
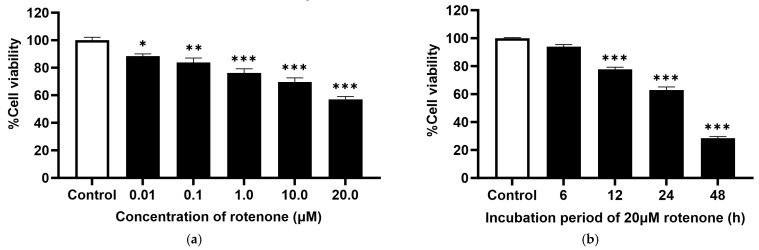
(**a**) Rotenone-induced neurotoxicity for 24 h in SH-SY5Y cells showed a dose-dependent reduction in cell viability, determined by an MTT assay. (**b**) 20 µM rotenone-induced neurotoxicity in SH-SY5Y cells was determined by MTT assay. Values are reported as the percentage of the untreated control and represent the mean ± SEM (*n* = 6). Significance was calculated using ANOVA [* = *p* < 0.05, ** = *p* < 0.01, and *** = *p* < 0.001] compared to the control.

The cytotoxicity of rotenone was investigated in SH-SY5Y cells. Cells were treated with various concentrations of rotenone (0.01, 0.1, 1.0, 10.0, and 20.0 μM) for 24 h. As shown in Figure 3a, the increasing concentration of rotenone resulted in a significant decrease in cell viability. The concentration of rotenone at 20 µM showed the greatest significant decrease in cell viability. Then this concentration of rotenone was used to investigate the effect of incubation periods (6, 12, 24, and 48 h). As shown in Figure 3b, a significant reduction in cell viability was observed when cells were incubated for a longer period when compared to the control group. The incubation with 20 µM rotenone for 24 h showed approximately the median lethal dose (LD_50_), reducing cell viability to 62.97 ± 2.21% of the control group. Therefore, 20 µM rotenone with an incubation period of 24 h was selected to determine the effects of DiAc-Cp-Mn on rotenone-induced neurotoxicity in SH-SY5Y cells. The incubation with 20 µM rotenone for 6 h did not significantly decrease cell viability or induce cytotoxicity. Then this condition was used in another subsequent experiment to determine the effect of interested compounds on cellular oxidative stress, such as intra-ROS levels, MMP, and antioxidant enzyme activities.

### 2.4. Effect of DiAc-Cp and DiAc-Cp-Mn on Rotenone-Induced Neurotoxicity in SH-SY5Y Cells

**Figure 4:**
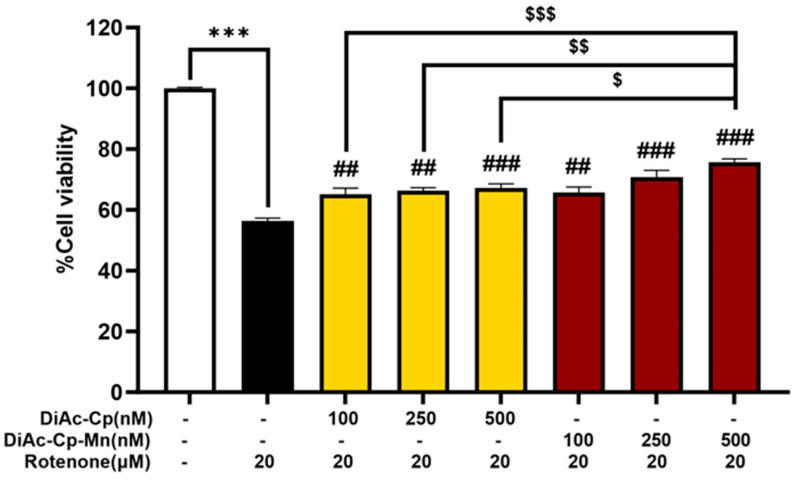
The neuroprotective effect of DiAc-Cp and DiAc-Cp-Mn against 20 μM rotenone-induced toxicity in SH-SY5Y cells for 24 h was determined by MTT assay. Values are reported as the percentage of the untreated control and represent the mean ± SEM (*n* = 6). Significance was calculated using a *t*-test [*** *p* < 0.001] compared to the control, an ANOVA [## = *p* < 0.01 and ### = *p* < 0.001] compared to the rotenone group, and an ANOVA [$ = *p* < 0.05, $$ = *p* < 0.01, and $$$ = *p* < 0.001] compared to the DiAc-Cp-Mn group.

DiAc-Cp and DiAc-Cp-Mn showed a protective effect against rotenone-induced toxicity in SH-SY5Y cells. Treatment with 20 μM rotenone for 24 h dramatically reduced cell viability to 56.40 ± 0.91% of the control group, compared to the control group. Pretreatment with DiAc-Cp and DiAc-Cp-Mn at concentrations of 100, 250, and 500 nM before rotenone exposure significantly increased cell viability, compared to the rotenone exposure group. The Mn complex had a higher protective potential than its parent compound. At a concentration of 500 nM, DiAc-Cp-Mn showed the most protective effect, increasing cell viability to 75.61 ± 1.15% of the control. The pretreatment of 500 nM DiAc-Cp-Mn demonstrated a significantly higher protective effect than the pretreatment of DiAc-Cp at concentrations of 100, 250, and 500 nM, which increased cell viability to 65.09 ± 2.04%, 66.24 ± 1.08%, and 67.26 ± 1.28% of the control, respectively (Figure 4). Since the protective effect of both DiAc-Cp and DiAc-Cp-Mn against rotenone-induced neurotoxicity showed a significant increase in cell viability concentrations of 500 nM, the concentrations of 500 nM of DiAc-Cp and DiAc-Cp-Mn were chosen for further experiments.

### 2.5. DiAc-Cp and DiAc-Cp-Mn Suppress Rotenone-Induced Intracellular ROS Overproduction in SH-SY5Y Cells

**Figure 5:**
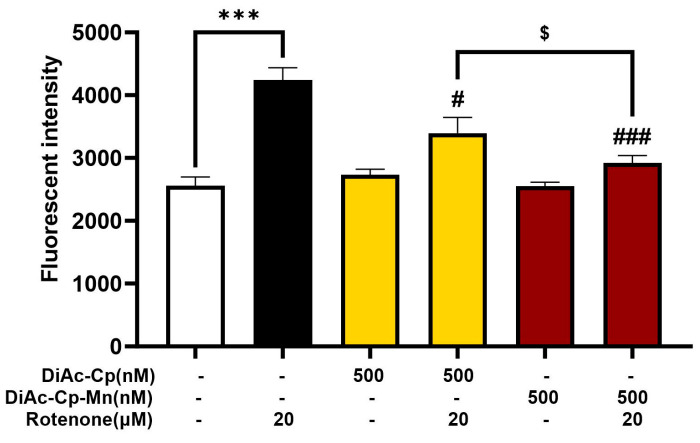
Levels of intracellular ROS in rotenone-treated SH-SY5Y cells were determined by the DCFH-DA method. Cells were pretreated with DiAc-Cp and DiAc-Cp-Mn before rotenone exposure for 6 h. Values are reported as the mean ± SEM (*n* = 6). Significance was calculated using a *t*-test [*** = *p* < 0.01] compared to the control, an ANOVA [# = *p* < 0.05 and ### = *p* < 0.001] compared to the rotenone group, and an ANOVA [$ = *p* < 0.05] compared to the DiAc-Cp-Mn group.

The presence of ROS is an important indicator of oxidative stress. DCFH-DA was used to detect the level of intracellular ROS in SH-SY5Y cells. As shown in Figure 5, treatment with 500 nM DiAc-Cp alone and 500 nM DiAc-Cp-Mn alone had no significant increase in intracellular ROS levels, while treatment with 20 μM rotenone for 6 h markedly increased ROS levels. Pretreatment with 500 nM DiAc-Cp and 500 nM DiAc-Cp-Mn before rotenone exposure significantly decreased the amount of intracellular ROS, compared to the rotenone group. Moreover, pretreatment with DiAc-Cp-Mn significantly diminished intracellular ROS, which was significantly greater than pretreatment with DiAc-Cp.

### 2.6. DiAc-Cp and DiAc-Cp-Mn Restored Mitochondrial Membrane Potential in Rotenone-Induced Mitochondrial Dysfunction in SH-SY5Y Cells

**Figure 6:**
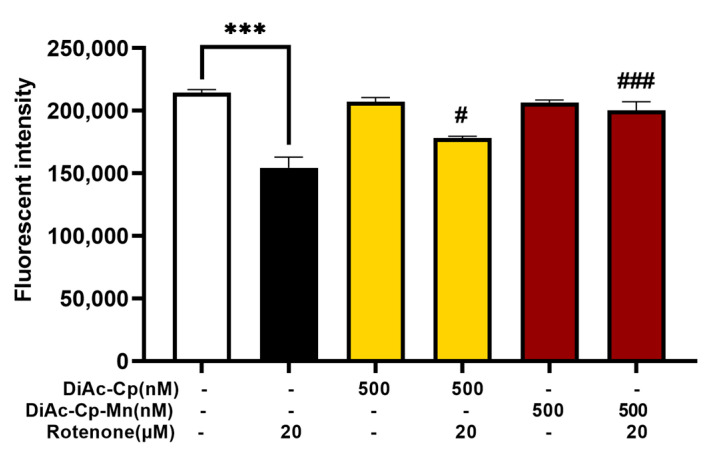
MMP in rotenone-treated SH-SY5Y cells was determined by the Rh-123 method. Cells were pretreated with DiAc-Cp and DiAc-Cp-Mn before rotenone exposure for 6 h. Values are reported as the mean ± SEM (*n* = 6). Significance was calculated using a *t*-test [*** = *p* < 0.001] compared to the control, an ANOVA [# = *p* < 0.05 and ### = *p* < 0.001] compared to the rotenone group.

The mitochondria are an important site of ROS formation and a target of ROS-induced damage. In this study, a mitochondrial-specific probe (Rh-123) was used in SH-SY5Y cells to assess MMP. Reductions in MMP were detected by weakening Rh-123 fluorescence intensities, indicating compromised mitochondrial function. As shown in Figure 6, treatment with 500 nM DiAc-Cp alone and 500 nM DiAc-Cp-Mn alone had no significant drop in MMP, while treatment with 20 μM rotenone for 6 h rapidly declined in MMP levels. Pretreatment with 500 nM DiAc-Cp and 500 nM DiAc-Cp-Mn before rotenone exposure significantly raised the amount of MMP, compared to the rotenone-treated group. Since pretreatment with DiAc-Cp-Mn escalated MMP, which was greater than pretreatment with DiAc-Cp. The Mn complex improved MMP levels and protected the cells from rotenone-induced mitochondrial dysfunction.

### 2.7. DiAc-Cp and DiAc-Cp-Mn Increased Antioxidant Enzyme Activity during Rotenone-Induced Oxidative Stress in SH-SY5Y Cells

**Figure 7:**
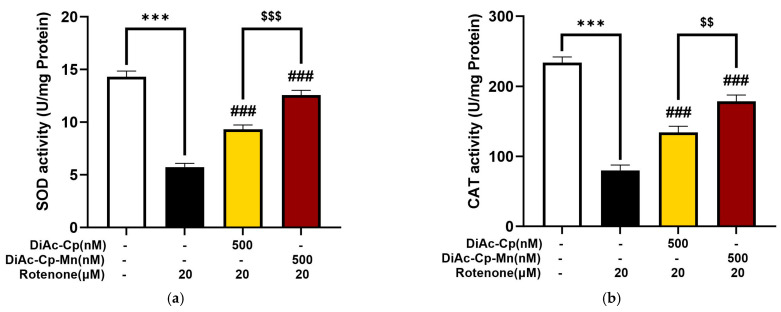
DiAc-Cp and DiAc-Cp-Mn mitigated rotenone-induced neurotoxicity on SH-SY5Y cells by improving (**a**) the SOD and (**b**) the CAT activities. Values are reported as the mean ± SEM (*n* = 6). Significance was calculated using a *t*-test [*** = *p* < 0.001] compared to control, an ANOVA [### = *p* < 0.001] compared to the rotenone group, and an ANOVA [$$ = *p* < 0.01 and $$$ = *p* < 0.001] compared to the DiAc-Cp-Mn group.

To understand the anti-oxidative effect of interested compounds, we investigated the activity of antioxidant enzymes, SOD, and CAT in DiAc-Cp and DiAc-Cp-Mn-treated SH-SY5Y cells under rotenone-induced oxidative stress. As shown in Figure 7a, the untreated group showed that the SOD activity was 14.30 ± 0.55 U/mg protein. Rotenone exposure for 6 h dramatically reduced the SOD activity to 5.73 ± 0.37 U/mg protein. Pretreatment with 500 nM DiAc-Cp and 500 nM DiAc-Cp-Mn before rotenone exposure significantly improved the SOD activity to 9.32 ± 0.41 and 12.58 ± 0.45 U/mg, respectively, compared to the rotenone group. Rotenone exposure also caused a reduction in CAT activity, as shown in Figure 7b. The untreated group showed that the CAT activity was 233.64 ± 8.12 U/mg protein. Rotenone exposure for 6 h markedly decreased the CAT activity to 79.99 ± 7.47 U/mg protein. Pretreatment with 500 nM DiAc-Cp and 500 nM DiAc-Cp-Mn before rotenone exposure significantly raised the CAT activity to 134.29 ± 8.55 and 178.62 ± 8.98 U/mg, respectively, compared to the rotenone group. These results indicated that DiAc-Cp-Mn had a more pronounced protective effect in enhancing SOD and CAT activity than DiAc-Cp significantly.

### 2.8. Effect of DiAc-Cp and DiAc-Cp-Mn on Gene Expression in Rotenone-Induced SH-SY5Y Cells

**Figure 8:**
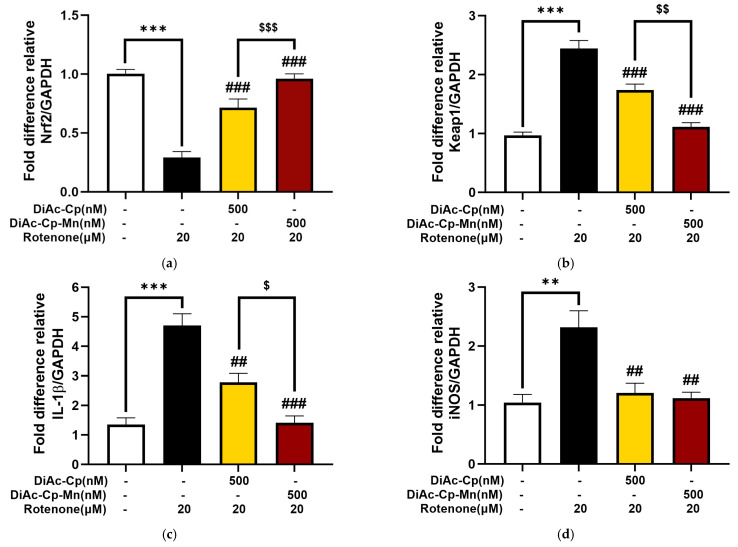
Effect of DiAc-Cp and DiAc-Cp-Mn on rotenone-induced expression of (**a**) NRF2, (**b**) KEAP1, (**c**) IL-1β, and (**d**) iNOS in SH-SY5Y cells. Values are reported as the percentage of the untreated control and represent the mean ± SEM (*n* = 6). Significance was calculated using a *t*-test [** = *p* < 0.01 and *** *p* < 0.001] compared to control, an ANOVA [## = *p* < 0.01 and ### = *p* < 0.001] compared to the rotenone group, and an ANOVA [$ = *p* < 0.05, $$ = *p* < 0.01, and $$$ = *p* < 0.001] compared to the DiAc-Cp-Mn group.

To explore the molecular mechanisms that might underlie the neuroprotective effect of DiAc-Cp-Mn, Nrf2/Keap1 (anti-oxidative defense regulation) and IL-1β/iNOS (inflammatory mediator) mRNA expressions were measured by q-PCR. Fold differences relative to the GAPDH housekeeping gene were used to quantify the expression levels. As shown in Figure 8, rotenone exposure significantly increased the expression of Keap1, IL-1β, and iNOS mRNA and decreased the expression of Nrf2 mRNA, compared to the control. Treatment of 500 nM DiAc-Cp and 500 nM DiAc-Cp-Mn before rotenone exposure significantly improved the expression of Nrf2, Keap1, IL-1β, and iNOS. Additionally, DiAc-Cp-Mn raised the expression of Nrf2 and diminished the expression of Keap1 and IL-1β more than DiAc-Cp.

## 3. Discussion

**Figure 9:**
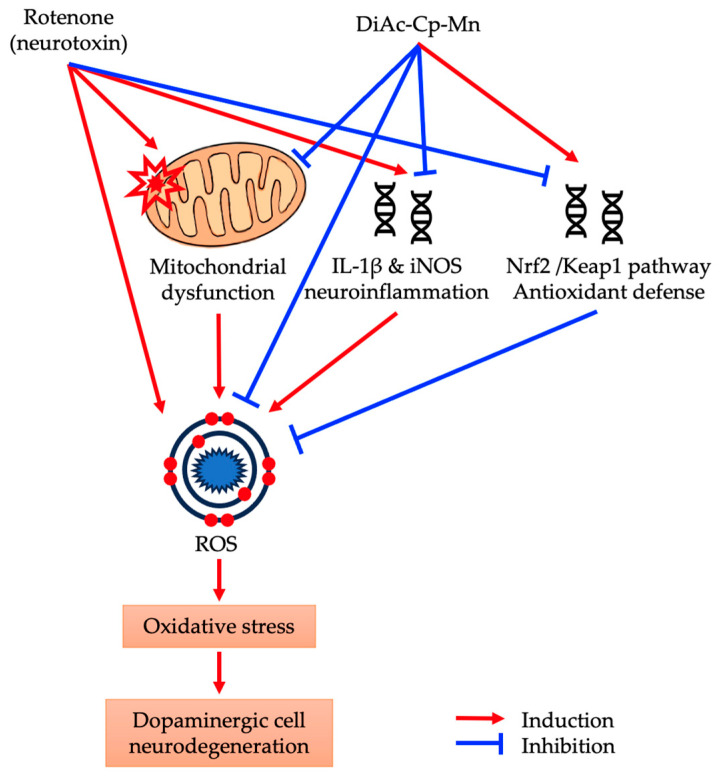
The neuroprotective effect of DiAc-Cp-Mn on rotenone-induced oxidative stress.

This study investigated the scavenging activities of a synthetic diacetylcurcumin manganese complex and diacetylcurcumin against the superoxide radical, hydroxyl radical, and hydrogen peroxide. Then, the neuroprotective effects of these compounds were evaluated using a rotenone-induced human dopaminergic neuroblastoma SH-SY5Y cell line model that mimics Parkinson’s disease. DiAc-Cp-Mn exhibited greater scavenging activity against superoxide radicals, hydrogen peroxide, and hydroxyl radicals than its parent compound. In cell-based assays, DiAc-Cp-Mn also showed greater neuroprotective effects against rotenone-induced neurotoxicity when compared to DiAc-Cp. Its underlying mechanism is involved in powerful antioxidant and anti-inflammatory activities by reducing intracellular ROS levels, restoring MMP, raising the antioxidant response, and inhibiting neuroinflammation (Figure 9).

Parkinson’s disease is a neurodegenerative disease. The main pathological features are progressive degeneration of dopaminergic neurons in the substantia nigra, mitochondrial dysfunction, neuroinflammation, and oxidative stress. Mitochondria perform a variety of tasks, including the generation of adenosine triphosphate (ATP), Ca^2+^ buffering, stress response, epigenetic signaling, and cell death pathways [14]. The generation of ROS from mitochondrial function is physiologic. To deal with these ROS, the body has two antioxidant systems, including enzymatic and non-enzymatic antioxidants, that neutralize the effects of these oxidants. For example, manganese superoxide dismutase (MnSOD) can convert superoxide to H_2_O_2_, and H_2_O_2_ is converted by GPx and CAT into water and oxygen. Accumulated evidence has reported decreased Complex I (NADH: ubiquinone oxidoreductase) activity and ubiquinone in the substantia nigra of PD patients [15,16,17]. Mitochondrial Complex I catalyzes the transfer of electrons from NADH to ubiquinone. The dysregulation of the electron transport chain causes extreme ROS formation and mitochondrial dysfunction, which are quite deleterious to neuronal cells due to an imbalance in the production of oxidants and antioxidant systems. A strategy to recover the balance between ROS formation and removal is a key to neuroprotection [18]. Administration of exogenous native antioxidant enzymes has not proven effective due to several pharmacokinetic limitations, antigenicity, and high production costs. The creation of low molecular weight SOD/CAT mimics is emphasized for pharmacological research as a means of overcoming these constraints.

Superoxide dismutase is an important antioxidant enzyme that plays a crucial role in scavenging superoxide radicals. There are two forms of SOD: cytosolic and extracellular Cu/Zn-SOD and mitochondrial Mn-SOD. Mn-SOD is particularly important in protecting mitochondria from oxidative damage [18,19]. Manganese ions in Mn-SOD undergo cyclic one-electron reduction and oxidation between Mn(II)/Mn(III) oxidation states as a result of the superoxide dismutation reaction. This redox shuffling of the active site manganese is dependent on two protons [20].

Mn^3+^ + O_2_^•−^ ↔ Mn^2+^ + O_2_

Mn^2+^ + O_2_^•−^ + 2H^+^ ↔ Mn^3+^ + H_2_O_2_

To mimic Mn-SOD activity, DiAc-Cp-Mn was synthesized by incorporating manganese (II) with the β-diketone part of two diacetylcurcumin molecules. Our current results demonstrated that DiAc-Cp-Mn (IC_50_ 5.69 ± 0.05 μM) exhibited greater superoxide radical scavenging activity than DiAc-Cp (IC_50_ 211.10 ± 4.27 μM), which is consistent with the previous reports that DiAc-Cp-Mn is a mimic of SOD [13]. Results suggest that DiAc-Cp-Mn can scavenge O_2_^•−^ with high efficacy and efficiency. Moreover, the superoxide dismutation, H_2_O_2_ is produced as a by-product, and it is one product of mitochondrial electron transport in aerobic respiration, Although H_2_O_2_ is less harmful than superoxide, controlling the amount of H_2_O_2_ is also critical. Interestingly, we also observed slightly reduced H_2_O_2_ levels (Table 1). DiAc-Cp-Mn also exhibited the CAT mimic by scavenging H_2_O_2_ with a lower IC_50_ (85.57 ± 0.32 μM) than its parent compound (IC_50_ 218.02 ± 3.00 μM). The efficiency of DiAc-Cp-Mn O_2_^•−^ elimination was higher than that of H_2_O_2_ reduction. DiAc-Cp-Mn is a mimic of both SOD and CAT. In addition, the HO· radical is extremely toxic and destroys any biological structure. The DiAc-Cp-Mn complex also exhibited greater hydroxyl radical scavenging activity when compared to its parent compound as well as trolox. The antioxidant activity of DiAc-Cp-Mn is attributed to the methoxy groups on the benzene rings and the manganese atoms at the active site. The redox reaction between manganese atoms and free radicals changes the oxidation state of the manganese atoms and the strong π electron donor ligand (the methoxy groups on the benzene rings), and the β-diketone system stabilizes the manganese complex at a high oxidation state [21]. Thus, the incorporation of a manganese atom at the active site of the antioxidant enhances SOD activity, CAT activity, and increases radical scavenging activity via the redox reaction of the manganese-centered atom. The molecular mechanism can be supported by our previous study using the electron paramagnetic resonance (EPR) technique. Curcumin manganese complex and DiAc-Cp-Mn decreased the EPR signal of the 5,5′-dimethyl-1-pyrroline-N-oxide (DMPO)/OOH and DMPO/OH adducts better than their parent compounds, curcumin and DiAc-Cp, respectively [22]. These findings suggest that the potent antioxidant properties of DiAc-Cp-Mn can be attributed to its ability to scavenge different types of ROS. It is in line with evidence from the previous study that Mn-SOD mimic, the manganese(III)tetrakis-(1-methyl-4-pyridyl)porphyrin (MnTm4PyP), has both SOD and CAT activities [23]. In addition, the DiAc-Cp-Mn complex (Mn-SOD mimic) exhibited stronger inhibition of lipid peroxidation than the diacetylcurcumin zinc complex (Zn-SOD mimic) and diacetylcurcumin copper complex (Cu-SOD mimic) [24]. This suggests that an Mn-SOD mimic could have a more potent protective effect against oxidative stress-induced neurodegeneration.

We then examined the neuroprotective effect of DiAc-Cp-Mn against rotenone-induced neurotoxicity in SH-SY5Y neuroblastoma cells. Rotenone, a neurotoxin, induces parkinsonism by inhibiting mitochondrial complex I on the respiratory electron transport chain, causing mitochondrial dysfunction and a decrease in the MMP [25,26]. These abnormalities cause ATP depletion and an increase in intracellular and mitochondrial ROS. The overproduction of ROS induces oxidative stress, triggers α-synuclein aggregation [10], increases proapoptotic Bcl-2 protein in the mitochondrial membrane [27], and ultimately contributes to the degeneration of dopaminergic neurons in the pathogenesis of PD. Several rotenone-induced cytotoxicity studies in SH-SY5Y cell models have been employed to explore the underlying mechanisms of action of putative PD treatments. In the present study, SH-SY5Y neuroblastoma cells were exposed to rotenone. The MTT assay was used to evaluate the dose and incubation periods of rotenone-induced about 50% cell death. The results obtained from the MTT assay indicated that the rotenone (20 μM) treatment for 24 h was a suitable condition for about 50% induction of cell death. The concentration of DiAc-Cp-Mn and its parent compound, with a concentration of up to 20 μM, had no cytotoxic activity in SH-SY5Y cells. In addition, DiAc-Cp-Mn exerted its neuroprotective effect against rotenone-induced cell death in a dose-dependent manner, and at the dose of 500 nM, DiAc-Cp-Mn exhibited a greater neuroprotective effect when compared to its parent compound. Therefore, this concentration was chosen as an effective dose for further studies.

Oxidative stress and mitochondrial dysfunction are the primary mechanisms of rotenone-induced degeneration of dopaminergic neurons [28]. Rotenone exposure induced the loss of MMP via mitochondrial complex I inhibition, leading to the opening of permeability transition pores and increasing intracellular ROS. With the increase of ROS in the dopaminergic neurons, redox homeostasis is lost, leading to antioxidant enzyme dysfunction, lipid peroxidation, neuro-inflammation, and neural cell damage, all of which contribute to the progress of neurodegeneration [24,29]. A promising approach that focuses on the inhibition of brain oxidative damage is the activation of the Nrf2 signaling pathway [30]. Nrf2 is a transcription factor that regulates antioxidative and anti-inflammatory pathways. Under normal conditions, Nrf2 is sequestered and stabilized by Keap1 in the cytoplasm. Keap1 inhibits the activation of Nrf2 and facilitates the proteasomes to degrade Nrf2. In the presence of oxidative stress, Nrf2 is released from Keap1. Nrf2 translocates to the nucleus and binds to the antioxidant-response element (ARE) to activate the transcription of antioxidant enzymes such as SOD, CAT, HO-1, GPx, and glutathione synthetase (GS) [31,32]. Inhibiting Nrf2-mediated transcription raises the vulnerability of dopaminergic neurons to oxidative stress [33]. Moreover, Nrf2 could suppress the inflammatory pathway through activating anti-inflammatory mediators (IL-10) and inhibiting proinflammatory cytokines (IL-1β, tumor necrosis factor-alpha (TNF-α), and interleukin-6 (IL-6)) [34]. To understand the molecular mechanism through which DiAc-Cp-Mn protects the neuron from rotenone exposure, the determination of MMP and intracellular ROS was performed. In addition, elevated ROS levels can reduce the activity of SOD and CAT, exacerbating oxidative stress [11]. Thus, the evaluation of antioxidant enzymes such as SOD and CAT activities and the function of the Nrf2/Keap1 signaling pathway were also measured to verify the regulation of oxidative stress. Firstly, we evaluated the effect of DiAc-Cp-Mn and DiAc-Cp on rotenone-induced MMP and enhancement of intracellular ROS. We found that DiAc-Cp-Mn exhibited the superior preventive effect against rotenone-induced MMP loss and enhancement of intracellular ROS when compared to its parent compound, suggesting that the neuroprotective effects of DiAc-Cp-Mn are mediated by the maintenance of mitochondrial function and reduction of intracellular ROS level. These findings are consistent with the results of in vitro radical-scavenging activities (Table 1). Moreover, we also demonstrated that rotenone treatment produced a reduction in SOD and CAT activities, which were related to the suppression of Nrf2 and the activation of Keap1 mRNA expression. DiAc-Cp-Mn pretreatment could elevate the activity of SOD and CAT to improve the capacity of cells to eliminate free radicals. These effects were accompanied by increased expression of Nrf2 and suppression of Keap1, leading to enhancement of the SOD and CAT activities. Interestingly, DiAc-Cp-Mn also exhibited better neuroprotective activities than DiAc-Cp. Therefore, this finding presented a novel perspective for the possible therapeutic use of DiAc-Cp-Mn in the PD model.

Additionally, rotenone-induced neuroinflammation by increases the levels of IL-1β, TNF-α, IL-6, and NO in SNpc [30]. The NF-κB signaling cascade is activated to intensify the inflammatory response. NF-κB is activated in the nucleus and binds to specific sites on DNA to regulate the expression of IL-1β, TNF-α, iNOS, and COX-2. The previous in vitro and in vivo studies showed that DiAc-Cp-Mn had strong NO radical scavenging activity [21]. DiAc-Cp-Mn protected against kainic acid-induced increases in NO levels, which cause neuronal cell death, in the rat hippocampus by their ability to scavenge NO and its antioxidative properties [11]. The previous reports showed that DiAc-Cp-Mn had anti-inflammatory activity by reducing the expression of COX-2 and iNOS on kainic acid-induced neurotoxicity in the rat hippocampus [12]. Consistent with the previous finding, the pretreatment with Mn complex attenuated rotenone-induced inflammation via downregulation of iNOS and IL-1β mRNA expression. The regulation of iNOS mRNA expression depends on the formation of a multiple intracellular signaling complex composed of Janus kinases, protein tyrosine kinases, protein kinase C, and mitogen-activated protein kinases, as well as transcription systems such as nuclear factor kB (NF-kB) and activator protein 1 (AP-1) [12]. The down-regulation of nuclear NF-κB, a major pro-inflammatory signaling protein, leads to decreased neuroinflammation [35]. SOD mimics mitigated neuro-inflammation by reducing these pro-inflammatory cytokines. Figure 9 depicts the putative multi-modes of action of DiAc-Cp-Mn against rotenone-induced oxidative damage.

## 4. Materials and Methods

### 4.1. Synthesis of Diacetylcurcumin Manganese Complex (DiAc-Cp-Mn)

Diacetylcurcumin (DiAc-Cp) and manganese(II)acetate were obtained from Angene International limited (Nanjing, China) and Sigma-Aldrich (St. Louis, MO, USA), respectively. DiAc-Cp-Mn was synthesized according to our previous report [22]. Manganese acetate (0.173 g, 1 mmol) was dissolved in 5 mL of ethanol (EtOH) and heated at 60–62 °C under nitrogen gas. DiAc-Cp (0.905 g, 2 mmol) was dissolved in a mixture of 25 mL ethyl acetate-methanol (7:3) and added dropwise to the manganese acetate solution. The reaction mixture was stirred and refluxed for 3 h under nitrogen gas. Then, DiAc-Cp-Mn was collected by filtration, washed with cold EtOH, and dried in a vacuum at room temperature over silica gel. A yellow-red powder of DiAc-Cp-Mn (0.744 g, 77.78% yield) was obtained with a mp of 198–201 °C. DiAc-Cp-Mn is stable to atmospheric oxygen and insoluble in alcoholic solvents, but soluble in DMSO. IR(KBr) (cm^−^^1^): 3500–3400 br. (O-H), 3010 (alkene C-H), 2940 (alkane C-H), 1762 (C=O ester), 1598 (C=O ketone), 1508 (C=C), 1416 (C=O enol, C=C (aromatic), 1297 (C-O phenol), 1216–1122 (C-O). ^1^H NMR (DMSO-d_6_): broad spectrum. Anal. Calc. for C_50_H_46_O_16_Mn (M.W. 957.22): C, 62.70; H, 4.84; Mn, 5.74. Found: C, 62.54; H, 4.76; Mn, 5.66.

### 4.2. Superoxide Radical Scavenging Activity Assay

In the PMS-NADH system, the oxidation of NADH generates superoxide radicals nonenzymatically. Superoxide radicals were assayed by their reduction of NBT. The reaction mixture (final volume was 200 µL) consisted of 50 µL of nitroblue tetrazolium (NBT) solution (258 µM NBT in 10 mM sodium phosphate buffer, pH 7.4), 50 µL of NADH solution (996 µM NADH in 10 mM sodium phosphate buffer, pH 7.4), and 50 µL of sample solution in DMSO at various concentrations. A 50 µL solution of phenazine methosulfate (PMS) (16.2 µM PMS in 10 mM sodium phosphate buffer, pH 7.4) was added to the mixture to initiate the reaction, which was then incubated for five minutes at 25 °C. The absorbance of the reaction mixture was examined at 562 nm against a blank determination. A drop in absorbance indicated a rise in the superoxide anion scavenging activity [36]. The percentage of superoxide radical scavenging was calculated according to Equation (1):

Superoxide radical scavenging activity (%) = (A_0_ − A_1_)/A_0_ × 100 (1)

where A_0_ represents the absorbance of the control and A_1_ represents the absorbance of the sample.

### 4.3. Hydroxyl Radical Scavenging Activity Assay

The hydroxyl radical scavenging activity was measured using the Fenton reaction, where the complexing agent EDTA reacts with ferrous ions and hydrogen peroxide to generate the hydroxyl radical. The hydroxyl radical degrades deoxyribose, and heating the products with thiobarbituric acid (TBA) at a low pH leads to the formation of malondialdehyde (MDA). MDA then reacts with TBA to form a pink MDA-TBA chromogen. The reaction mixture contained 108 µL of 10 mM deoxyribose, 30 µL of 1 mM EDTA, 60 µL of sample solution in 5% *w*/*v* tween80 at various concentrations, 3 µL of 10 mM ferric chloride, 99 µL of 50 mM phosphate buffer (pH 7.4), and 30 µL of 10 mM hydrogen peroxide. The reaction was started by adding 30 µL of 1 mM ascorbic acid. The mixture was incubated at 37 °C for 60 min. Afterward, 360 µL of 0.5% *w*/*v* TBA and 360 µL of 10% *w*/*v* trichloroacetic acid (TCA) were added, followed by heating at 100 °C for 15 min. The absorbance of the reaction mixture was measured at 532 nm at room temperature [31]. The percentage of hydroxyl radical scavenging was calculated according to Equation (2):

Hydroxyl radical scavenging activity (%) = (A0 − A1)/A0 × 100(2)

where A_0_ is the absorbance of the control and A_1_ is the absorbance of the sample or the standard.

### 4.4. Hydrogen Peroxide Scavenging Activity Assay

The hydrogen peroxide scavenging activity was measured by the reaction of ferrous ions with 1,10-phenanthroline to generate an orange-colored tri-phenanthroline complex. The oxidation of Fe(II) to Fe(III) by hydrogen peroxide leads to the non-complexation of 1,10-phenanthroline. The presence of ROS scavengers reduces the effect of hydrogen peroxide, increasing the formation of the orange-colored tri-phenanthroline complex [33]. The reaction mixture contained 75 µL of 1 mM ferrous ammonium sulfate, followed by 450 µL of sample solution in ethanol at various concentrations and 19.5 µL of 5 mM H_2_O_2_. The reaction mixture was incubated in the dark at 25 °C for 5 min. Then 450 µL of 1 mM 1,10-phenanthroline was added to the mixture. The reaction mixture was shaken and incubated at 25 °C for 10 min. The absorbance of the reaction mixture was measured at 510 nm [37]. The percentage of hydrogen peroxide scavenging was calculated according to Equation (3):

Hydrogen peroxide scavenging activity (%) = A_1_/A_0_ × 100 (3)

where A_0_ is the absorbance of the control and A_1_ is the absorbance of the sample or the standard.

### 4.5. Neuronal Cell Culture

The human neuroblastoma SH-SY5Y cell line, ATCC-CRL 2266 (A.N.H. Scientific Marketing Co., Ltd., Bangkok, Thailand), as an in vitro model of dopaminergic neurons for Parkinson’s disease, was grown in DMEM (Dulbecco’s modified Eagle’s medium, Sigma-Aldrich, St. Louis, MO, USA) with 10% fetal bovine serum (FBS) and antibiotic-antimycotic (100 U/mL penicillin, 100 μg/mL streptomycin, and 2.5 μg/mL fungizone) solutions. Cultures were kept at 37 °C in a humidified incubator with 5% CO_2_ and 95% air. Every two days, the cell culture media was changed.

### 4.6. Cell Viability Assay

MTT [3-(4,5-dimethylthiazol-2-yl)-2,5-diphenyltetrazolium bromide] (Invitrogen^TM^, Eugene, OR, USA) was used for assessment of cell viability. MTT is reduced to purple formazan by mitochondrial enzymes. About 5 × 10^4^ SH-SY5Y cells/100 µL were seeded per well in a 96-well culture plate and incubated for one day in the presence of 5% CO_2_ at 37 °C. After treatments were performed, the cells were incubated with 0.5 mg/mL MTT (100 µL) for 2–4 h at 37 °C. The medium was removed carefully, and then the purple formazan crystals were dissolved in DMSO (100 µL). The absorbance was measured by a spectrophotometer at 570 nm using a microplate reader (PerkinElmer, Inc., Shelton, CT, USA) [38]. The percentage of cell viability was calculated according to Equation (4):

Cell viability (%) = (A_1_/A_0_) × 100 (4)

where A_0_ is the absorbance of the control and A_1_ is the absorbance of the sample and the standard.

#### 4.6.1. Effect of DiAc-Cp and DiAc-Cp-Mn on Neuronal Cell Viability

To determine the cytotoxicity of DiAc-Cp and DiAc-Cp-Mn, SH-SY5Y cells were incubated with various concentrations (0.01, 0.1, 1.0, 10.0, and 20.0 µM) for 24 h. MTT assays were used to determine cell viability.

#### 4.6.2. Rotenone-Induced Cytotoxicity in SH-SY5Y Cells 

To evaluate the cytotoxicity of rotenone, SH-SY5Y cells were incubated with different concentrations of rotenone (0.01, 0.1, 1.0, 10.0, and 20.0 μM) for 24 h. To optimize the incubation period for rotenone-induced toxicity, SH-SY5Y cells were incubated with an optimal concentration of rotenone for 6, 12, 24, and 48 h. MTT assays were used to determine cell viability. The optimal condition of rotenone-induced neurotoxicity was used in subsequent experiments.

#### 4.6.3. DiAc-Cp and DiAc-Cp-Mn Treatment against Rotenone-Induced Neurotoxicity

MTT assays were used to assess the neuroprotective effect of DiAc-Cp and DiAc-Cp-Mn on rotenone-induced neurotoxicity. SH-SY5Y cells were pretreated with DiAc-Cp or DiAc-Cp-Mn for 4 h before rotenone (Table 2). The effective concentrations of both compounds were used to investigate the protective effect in other experiments.

### 4.7. Measurement of Intracellular Reactive Oxygen Species

The accumulation of intracellular ROS was measured by spectrofluorometry, using 2,7-dichloro-dihydrofluorescein diacetate (DCFH-DA) (Invitrogen^TM^, Eugene, OR, USA). Non-fluorescent DCFH-DA is converted to DCFH by cellular esterase, and DCFH is oxidized by ROS to form fluorescent dichlorofluorescein (DCF). After pretreatment for 4 h with DiAc-Cp-Mn or DiAc-Cp following incubation with 20 µM rotenone for 6 h, SH-SY5Y cells were incubated with DCFH-DA at a concentration of 20 µM for 30 min at 37 °C. After washing twice with PBS, the fluorescence intensity was measured at 488 nm (excitation) and 525 nm (emission) using a fluorescence microplate reader [39].

### 4.8. Measurement of Mitochondrial Membrane Potential

MMP was assessed by spectrofluorometry using rhodamine 123 (Rh-123) (Invitrogen^TM^, Eugene, OR, USA), which is a fluorescent mitochondria-targeting dye. Depolarization of the mitochondrial membrane results in the loss of Rh-123 and intracellular fluorescence reduction. After pretreatment for 4 h with DiAc-Cp-Mn or DiAc-Cp following incubation with 20 µM rotenone for 6 h, SH-SY5Y cells were incubated with Rh-123 at a concentration of 10 µM for 30 min at 37 °C. After washing twice with PBS, the fluorescence intensity was measured at 485 nm (excitation) and 530 nm (emission) using a fluorescence microplate reader [39].

### 4.9. Measurement of Antioxidant Enzyme Activities

SOD catalyzes the dismutation of O_2_^•−^ to H_2_O_2_ and O_2_. The catalytic breakdown of hydrogen peroxide into water and oxygen is catalyzed by CAT. Cells can be protected against oxidative damage by SOD and CAT [18]. The activities of SOD and CAT in SH-SY5Y cells were determined using the commercial SOD19160 Assay Kit and the CAT100 Catalase Assay Kit. (Sigma-Aldrich, St. Louis, MO, USA), respectively. For the SOD activity assay, after pretreatment for 4 h with DiAc-Cp-Mn or DiAc-Cp following incubation with 20 µM rotenone for 6 h, SH-SY5Y cells were gently trypsinized and transferred to an ice-cold tube. The cells were centrifuged at 250× *g* for 10 min at 4 °C, and the supernatant was discarded. Then, the cell pellets were washed with ice-cold PBS and homogenized in 0.5–1.0 mL of cold PBS per 100 mg of cells. The cells were centrifuged at 1500× *g* for 10 min at 4 °C. The supernatant was then collected for assaying. For the CAT activity assay, after pretreatment for 4 h with DiAc-Cp-Mn or DiAc-Cp following incubation with 20 µM rotenone for 6 h, SH-SY5Y cells were gently dislodged with a rubber policeman and transferred to a tube on ice. The cells were centrifuged at 250× *g* for 10 min at 4 °C and the supernatant was discarded. The cell pellets were washed with ice-cold PBS and homogenized in 1.0–2.0 mL of cold 1× assay buffer per 100 mg of cells. The cells were centrifuged at 10,000 rpm for 15 min at 4 °C. The supernatant was then collected for assaying. The total protein concentration in the sample, as determined by the Bradford method, was used to normalize all antioxidant enzyme activity. SOD and CAT activities are expressed as U/mg protein [40].

### 4.10. Quantitative Real-Time Polymerase Chain Reaction (q-PCR)

The mechanisms of DiAc-Cp and DiAc-Cp-Mn on the etiology of antioxidant and anti-inflammatory effects were investigated using q-PCR. Total RNA was extracted from SH-SY5Y cells with TRIzol^®^ reagents (Invitrogen^TM^, Thermo Fisher Scientific, Waltham, MA, USA). Chloroform and isopropanol were added for phase separation and RNA precipitation, respectively. Complementary DNA (cDNA) was synthesized from total RNA using oligo(dT) primers and Superscript^TM^ III reverse transcriptase (Invitrogen^TM^, Thermo Fisher Scientific, Waltham, MA, USA) via FlexCycler^2^ PCR Thermal Cycler (Analytik Jena, Jena, Germany). In the denaturation step, primers were specifically attached to single-stranded DNA (ssDNA) to start extension in the annealing step. The non-specific fluorescent dye technique, SYBR^®^ green supermixes (Bio-Rad, Hercules, CA, USA), was used to determine double-stranded DNA (dsDNA). Real-time q-PCR was performed on the Bio-Rad CFX Connect^®^ Real-Time System (Bio-Rad, Hercules, CA, USA). Glyceraldehyde-3-phosphate dehydrogenase (GAPDH) was used as a reference gene. The data on gene expression was presented in fold-difference relatives, which were then calculated using the 2^−∆∆Ct^ method [41]. The following primers were used and synthesized by Macrogen (Seoul, Republic of Korea); GAPDH 5′-AAC ACA GTC CAT GCC ATC AC-3′ (sense) and 5′-TCC ACC ACC CTG TTG CTG TA-3′ (antisense); IL-1β 5′-GAC AGC AAG TGA TAG GCC-3′ (sense) and 5′-CGT CGG CAA TGT ATG TGT TGG-3′ (antisense); iNOS 5′-AGA AGG AAA TGG CTG CAG AA-3′ (sense) and 5′ GCT CGG CTT CCA GTA TTG AG-3′ (antisense) [42]; Nrf2 5′- CAG TGC TCC TAT GCG TGA A-3′ (sense) and 5′ GCG GCT TGA ATG TTT GTC-3′ (antisense) [43]; Keap1 5′- CAT CCA CCC TAA GGT CAT GGA-3′ (sense) and 5′ GAC AGG TTG AAG AAC TCC TCC-3′ (antisense) [32].

### 4.11. Statistical Analyses

In vitro studies are presented as the mean ± standard deviation (SD). In cell-based analysis, results are expressed as the mean ± standard error of the mean (S.E.M.) for each group. The untreated group and the rotenone-induced group were analyzed by an unpaired *t*-test. Multiple comparisons among the treatment groups were determined using the one-way analysis of variance (ANOVA) with a post hoc Tukey. The significant difference was considered at *p* < 0.05. The software IBM^®^ SPSS^®^ Statistics version 28.0 was used for data analysis.

## 5. Conclusions

The potential benefits of DiAc-Cp-Mn as a therapeutic compound for neurodegenerative disorders, specifically Parkinson’s disease, are based on its protective effects against oxidative stress. DiAc-Cp-Mn has been found to have a greater protective effect compared to diacetylcurcumin, primarily due to its radical-scavenging activities and mimicking of SOD and CAT properties. DiAc-Cp-Mn has shown efficacy in reducing intracellular ROS levels and maintaining MMP in the rotenone-induced group. The complex also elevated the activity of SOD and CAT to improve the capacity of cells to remove free radicals. These effects were accompanied by the increased expression of Nrf2 and the suppression of Keap1. These effects contribute to a decrease in oxidative damage, which is believed to be a significant factor in neurodegenerative disorders. Moreover, the complex has been found to significantly decrease the expression of pro-inflammatory cytokines such as IL-1β and iNOS. The parent compound, diacetylcurcumin, did not exhibit the same reduction in IL-1β expression. DiAc-Cp-Mn could be a promising therapeutic compound for neurodegenerative disorders, particularly Parkinson’s disease, due to its ability to reduce oxidative stress. However, further studies are needed, particularly in vivo studies, to investigate the effects of the compound on behavioral disorders and symptoms associated with neurodegenerative disorders. The findings of this study indicate that DiAc-Cp-Mn seems to be a promising neuroprotective agent for neurodegenerative diseases such as Parkinson’s disease, in which oxidative stress and neuroinflammation have been implicated.

## Figures and Tables

**Table 1:** IC_50_ of DiAc-Cp and DiAc-Cp-Mn for various antioxidant systems.

	Scavenging Activity Assay (IC_50_)		
Sample	Superoxide Radical	Hydrogen Peroxide	Hydroxyl Radical
DiAc-Cp (µM)	211.10 ± 4.27	218.02 ± 3.00	46.94 ± 1.65
DiAc-Cp-Mn (µM)	5.69 ± 0.05	85.57 ± 0.32	15.14 ± 0.19
SOD (unit/mL)	5.90 ± 0.20	-	-
Trolox (µM)	-	1118.18 ± 15.14	99.78 ± 14.26

Each value in the table is represented as the mean ± SD (*n* = 3).

**Table 2:** Measurements of neuroprotective effects of DiAc-Cp and DiAc-Cp-Mn on rotenone-induced toxicity.

Treatment	Concentration (µM)	Duration (h)
DiAc-Cp/Rotenone	0.1, 0.25, and 0.5/20.0	4/24
DiAc-Cp-Mn/Rotenone	0.1, 0.25, and 0.5/20.0	4/24

## Data Availability

Data are contained within the article.
